# Medical News and Miscellany

**Published:** 1887-07

**Authors:** 


### Medical News and Miscellany.

Dr. D. Dupree, late of this city, Austin, has removed to Dallas, to practice. We commend the Doctor to the good offices of the professional brethren, and wish him success.

MARRIED.

Miss Stella Wooten, daughter of Dr. T. D. Wooten, President Board of Regents State University, was married on 22nd of June ult., to Mr. W. J. Bailey, of Fort Worth, Texas.

Dr. Bryce, Editor of *Southern Clinic*, Richmond, Va., devotes his editorial space in July issue, to a defense of himself against charges published in the newspapers against him, by some one connected with the Virginia Medical College, whom he had offended. The Doctor proves his case very conclusively, to us.

Dr. E. W. Walton, of Georgetown, Texas, son of our townsman, Col. Buck Walton, has recently been elected Assistant House Surgeon to the Manhattan Eye and Ear Infirmary, in New York. Dr. Walton went on last fall to take a special course at the New York Post Graduate Medical College. It is expected that he will return to Texas in two or three years.

Dr. O. F. Kendall, late of Throckmorton, (a subscriber to the Journal), came to Austin and located. After remaining a few weeks, during which time he made a good impression and many friends, and purchased a handsome turn out, he left suddenly, and to his friends, quite unexpectedly, and returned to his old home. We regret that the Doctor left, for we believe, he would have done well, had he stuck it out awhile.

An Extraordinary Case of Dry Gangrene, following phlegmasia dolens, occurred recently in Dr. Swearingen's practice, in a primi para in Austin, in the higher social ranks. The labor was normal in all respects, and the occurrence of the phlegmasia was unexpected. It was thought to be the result of emboli of some of the popliteal vessels. Sloughing of the entire right foot took place, and the bones up to the astragalus were disarticulated. Her recovery, under the circumstances, was most extraordinary, she being a delicate and tenderly raised young lady, who was not thought to possess such remarkable vital powers. We hope to give our readers the clinical history of the case, as it stands so far as we know, unparalleled in medical literature.

---

To Subscribers.—We know that the times are hard, and money in the country, scarce. While we will be pleased at all times to receive remittances for subscriptions, it is not, by any means, a *condition* to continuing the Journal. If any find it inconvenient to remit at present, and desire to continue the Journal, we will take ever so much pleasure in extending all the time they want, to pay for it. At the close of the Journal year, we made out and mailed all bills; and again, at the beginning of the new volume. This is simply *business*, and no one should consider himself specially *dunned*. If convenient, remit: if not, drop a card, and you shall have all the indulgence you want. We do not want any patron to stop the Journal simply because he cannot pay for it in advance, or even at the end of the Journal year. While we are not Bondholders, we are able, and more than willing, to carry our subscribers over another year—such as desire it. Send along your names, for the new volume just beginning with this number.

---

A Sample Mail. — The following are extracts from letters received by *one mail* (Sunday p. m., July 17); in addition, there were nine other remittances without comment. This will give some idea of the estimation in which the Journal is held by the better element—the educated and liberal minded members of the profession. That our efforts in behalf of organization are thoroughly appreciated, and are telling, we call attention to the fact that the prime movers in nearly all the recent county organizations are staunch supporters of, and subscribers to the Jour-

NAL. This may be better understood by comparing the present size and number of medical organizations in Texas, with the status three years ago.

FLATONIA, July 14.

"I like our Texas representative very much;" find enclosed, etc.

B. W. BRISTOW.

BAIRD, July 16.

"I am receiving the JOURNAL regularly, and like it very much; enclosed please find, etc."

D. J. WILSON.

MARLIN, July 16.

"I take pleasure in renewing my subscription to your very interesting JOURNAL for another year. Enclosed, etc."

R. C. NETTLES.

——— TEXAS, July 15.

"My wife, as well as myself, read the JOURNAL with a great deal of pleasure."

J. W. S.

CORSICANA, July 14.

"It affords me much pleasure to enclose herewith, my subscription for another year. Will send contribution shortly."

W. CASTON.

KILDARE, July 15.

"Enclosed find P. O. M. Order ($2), for which send me the JOURNAL for another year. *May it always flourish.*"

T. W. CONNERLY.

DELRIO, July 15.

"I beg leave to offer my sincere congratulation on the continued success of the JOURNAL. I enclose P. O. M. Order to renew my subscription.

E. G. NICHOLSON.

BOSQUEVILLE, July 14.

"I enclose $2; my subscription for another year. I like the JOURNAL very much. *I would not be without it.*"

R. F. MINNOCK.

ELPASO, July 15.

"The JOURNAL gets better and better all the time. Enclosed please find, etc."

J. T. HARRINGTON.

THE TEXAS AND SOUTHWESTERN DRUGGIST, Waco, Texas, is one of our sprightliest exchanges. It is gotten out in good style, and is doing good service in organizing the druggists of Texas, and advocating a higher pharmacy. The *Druggist* is advocating a college of pharmacy in Texas. Success to it.

---

### THE TREATMENT OF INTERNAL HÆMORRHOIDS IN SITU.

Dr. Q. A. Shuford, of Tyler, Texas, has invented an anal speculum, a cut and description of which are given in our advertising pages, which he claims is peculiarly adapted to use in the treatment of internal hæmorrhoids. Instead of causing the patient to strain till the tumor or tumors are extruded beyond the anal sphincter, or dragging them out by means of tenaculum, either of which process causes pain, and afterwards congestion and soreness, it is claimed, that when the speculum is introduced, and the plug withdrawn the tumors are exposed and can be injected *in situ*, by means of a hypodermic with a long needle made especially for the purpose. Dr. Shuford claims good results from the following formulæ, thus used:

℞ Glycerole of Boracic Acid ʒiv

" " Salicylic " ʒiv

Carbolic Acid pure ʒiii Mix: rub thoroughly in a mortar. Let stand in vial till it clears.

After a thorough evacuation of the lower bowels the speculum is introduced, oiled, of course, and three to five drops of the mixture are to be injected into small, and five to eight drops into large hæmorrhoids. The center of the pile should be chosen for the insertion of the needle, and the fluid should be slowly injected, the point being turned about if it be a large tumor. "The tumor will become atrophied, shrink up, and peel off," says the doctor, "about the fourth or fifth day," He recommends that the injection be repeated if necessary, every eight or ten days; meantime the patient should bathe the part freely in hot or cold water, and use such emolient applications as will relieve any resultant soreness. It is claimed for this method that the treatment does not interfere with business—the patient being up and about as usual.

Dr. McLaughlin informs us that while at the Polyclinic in New York recently, Prof. Wyeth called his attention to the above formula which was hung on the wall in print, informing him that it was recommended and used by Dr. Shuford of Texas, and that it was in daily use in the Polyclinic, with good results, and recommended the class to take a memorandum of it.

---

Our distinguished townsmen, Drs. T. J. Tyner and T. D. Wooten, will attend the International Congress, and Dr. Wooten has already gone north. Dr. Tyner will leave August 1, to attend a special course at the N. Y. Post Graduate Medical College, prior to the meeting of the Congress.

---

Dr. Frank Litten, of Austin, who graduated at Jefferson Medical College, Philadelphia, last session, and who remained until uow in the Hospital there, has returned, and will engage in practice with his father, Dr. J. M. Litten, one of the old residenters of Texas.

---

YELLOW FEVER.—The latest advices give (July 14) 110 cases and 30 deaths from yellow fever at Key West. Hospital steward Wells has recovered. It was reported that the fever had made its appearance at Memphis, but Dr. Thornton, President Board of Health, officially denies the report.

---

RESIDENT STUDENTS IN THE CHARITY HOSPITAL AT NEW ORLEANS. Under the laws of Louisiana, none but Louisiana students are eligible to appointment as resident students in this noble Charity. At last meeting of the Louisiana State Medical Association, Dr. Jos. Jones introduced a resolution that the State Medical Association memorialize the General Assembly to change the law, throwing the doors open to all students by competitive examination.

---

### A MEDICAL WORK BY A TEXAS PHYSICIAN.

Dr. B. E. Hadra, of Austin, has written a work "Lesions of the Pelvic Floor," which is being published as a serial in the *Medical Register*. When completed, it will appear in book form. It is profusely illustrated. We look to see much credit awarded the Doctor for the work: it possesses much merit in the way of *originality*, a rare quality these days, and is a reflex of his own personal experience.

Dr. R. M. Swearingen is taking a very active part in the crusade against intemperance, and has made some telling speeches in favor of the prohibition amendment to the constitution. At Austin, and at Belton, the Doctor was greeted with the largest and most enthusiastic audiences of ladies that ever turned out to hear any one speak, a compliment which he much appreciates.

---

DELICATE SENSIBILITIES.—It seems most extraordinary that some physicians, men who have character and standing, will *receive* a Medical Journal month after month, *for a year or more*, and when a mild and courteous intimation is given, that it takes *money* to publish such Journal, will *resent* it as an *insult*, and very curtly reply: "I never subscribed."

How delightful, by contrast, the kind words of *appreciative* readers, as shown in the few extracts elsewhere given: and the following, which has since been received, from a stranger(?)

COLUMBUS, July 21, 1887.

*My dear Sir and Friend:*

It seems strange to me, that instead of your dunning me, I have to dun you for my bill. However, enclosed find P. M. O. Order for amount due to date, and if you please, continue my subscription.

With the kindest wishes for your health, continued usefulness and welfare, I remain, truly your friend,

J. M. B———

---

WE notice that the Kentucky State Medical Society adopted at its last session the following:

*Resolved*, That the recent published attack upon the personal reputation of Dr. Dudley S. Reynolds is unfounded and unjust.

*Resolved*, That the Kentucky State Medical Society in regular session assembled, bear testimony to the high professional and social standing of Dr. Reynolds, and repel the aspersions against a physician who for fifteen years has ranked among its foremost members."

And has it come to this that even so classical an exponent of the good, the true and the beautiful as Dudley S. Reynolds, must needs suffer aspersion and calumniation?

*St. Louis Weekly Review.*

